# Quarterly Report of the Obstetric Department of the Philadelphia Dispensary, for First, Second and Third Months, 1843

**Published:** 1843-05-13

**Authors:** Joseph Warrington

**Affiliations:** Obstetric Physician


### QUARTERLY REPORT
### OF THE
### OBSTETRIC DEPARTMENT OF THE PHILADELPHIA DISPENSARY,
*For First, Second, and Third Months*, 1843.

By Joseph Warrington, Obstetric Physician.

Forty-four women have been delivered, all at or near the full term of utero-gestation except one, in which the ovum having been developed to the sixth month, was expelled at the termination of the seventh.

The average duration of labour, in thirty-nine cases, was fifteen hours, twenty-six minutes; the extremes being one and a half, and sixty-four hours.

The average amount of time required for the spontaneous delivery of the placenta, in thirty-seven cases, was ten minutes; the extremes being five, and sixty minutes.

Manual assistance was afforded for the delivery of this mass in three instances, in which the disk presented so large a surface to the orifice of the uterus that it could not pass through it until one edge was drawn down by the finger after the patient had made ineffectual effort for extending it, in one instance fifteen minutes, another at twenty-five, and the third at forty-five minutes subsequent to the expulsion of the fetus.

Forty-five children have been delivered, one woman having twins.

Twenty-nine of these children were males, and sixteen were females.

The vertex of the cephalic extremity of the fetal ellipse presented in thirty-two instances; of these, nineteen were in the first position, and seven in the second.

One child, developed to the sixth month, but born at the end of the seventh, presented the breech in the fifth position.

One of the doublets presented the dorsum; it was converted by the use of a hand into the second position of the breech.

The umbilical cord was noted in one case to be about one-half the usual length, measuring ten inches.

One child had a slight deformity of the pedal articulation, for the correction of which appropriate dressings have been applied.

One child also had exomphalos, which is improving rapidly under a compress and roller.

The os uteri in one case, during the first stage of labour, continued so rigid as to render free bleeding necessary to promote relaxation.

There were two instances of irregular contractions of the uterus for about two weeks before the termination of labour; in one of these the os uteri was observed to be partially dilated about eight days previous to delivery.

In the second stage of the labour, the entire duration of which has been noted to be sixty-four hours, forty grains of ergot were administered, without, however, any marked effect in increasing the expulsive action of the uterus.

The forceps was used in one case to deliver from the superior strait of the pelvis a child, the occipito-mental diameter of which measured seven, the occipito-frontal six, the biparietal three and a half, and occipito-bregmatic three and a half inches.

This instrument was used also in a primiparous female, for the delivery of a child, the head of which had become arrested at the inferior strait. The child weighed ten and a half pounds.

There were three instances of hemorrhage after delivery. Free friction, regular compression, and the ergot, in tincture or substance, were resorted to. The flow became speedily arrested in two cases; in the third, however, the atony of the uterus was so great, that its contractions could not be reproduced until the hand had been introduced to stimulate its internal surface. Recovery was rapid in the two first instances; but in the third the patient suffered much from the usual consequences of excessive loss of blood for several days.

There were five cases of attacks of inflammation of the peritoneum, uterus or its appendages, after delivery. In three of them the disease was quickly arrested by prompt and free venesection, cathartics, and anodyne diaphoretics. One continued through a number of days, though actively depleted, both generally and locally, and one resisted the action of all treatment.

The patient was badly accommodated in one of the densely inhabited houses on the bank side of Front street, and had suffered much from moral and physical causes, both before and subsequent to her parturition.

She died on the sixth day after delivery.

All the other women recovered.

Nineteen of the cases above reported have been attended by nurses in the employment of the Nurse Charity Society; and we continue to be persuaded that much benefit is conferred upon most of those who derive such aid.

